# Syncope and Sarcoidosis: A Criss-Cross of Diagnoses

**DOI:** 10.7759/cureus.16367

**Published:** 2021-07-13

**Authors:** Swetha Ramireddy, Ali Shakir

**Affiliations:** 1 Internal Medicine, Henry Ford Macomb Hospital, Clinton Township, USA; 2 Cardiology, Henry Ford Macomb Hospital, Clinton Township, USA

**Keywords:** noncaseating granulomas, cardiac sarcoidosis, syncope, sarcoidosis, presyncope, bifascicular block

## Abstract

Syncope has a broad range of differential diagnoses. Sarcoidosis, a multisystem inflammatory disorder characterized by the formation of noncaseating granulomas, is a rare but important diagnosis to consider while evaluating patients presenting with presyncopal or syncopal symptoms. Although sarcoidosis is most commonly known to affect the lungs, it is estimated that at least 25% of patients with sarcoidosis have myocardial involvement, with only 5% of these patients showing clinical symptoms. Here, we present the rare case of a Caucasian male patient diagnosed with cardiac sarcoidosis after presenting to the hospital with presyncope. The patient had an internal cardioverter-defibrillator placed during hospitalization and was initiated on prednisone and methotrexate in the outpatient setting. He exhibited clinical and radiographical improvement in the six-month follow-up period after treatment was initiated.

## Introduction

Cardiac sarcoidosis is a rare but important diagnosis to consider while evaluating a patient presenting with presyncope or syncope. Characterized by the formation of noncaseating granulomas within the heart, cardiac sarcoidosis is a disease associated with a high degree of morbidity and mortality if the diagnosis is delayed. The clinical presentation of patients with cardiac sarcoidosis is variable. Patients may range from being asymptomatic or having nonspecific complaints such as chest pain and shortness of breath to presenting with malignant arrhythmias, heart failure, and conduction abnormalities that may lead to presyncope and syncope [1-5]. Therefore, although reflex mediated is the most common cause of syncope, clinicians should keep cardiac sarcoidosis on the differential in patients presenting with presyncope or syncope with an otherwise unexplained baseline electrocardiogram (EKG) abnormality, as these may be the only cues for diagnosis [1,6]. Here, we present a particularly uncommon case of a Caucasian male who was diagnosed with cardiac sarcoidosis after presenting to the hospital with a presyncopal event.

## Case presentation

**Figure 1:**
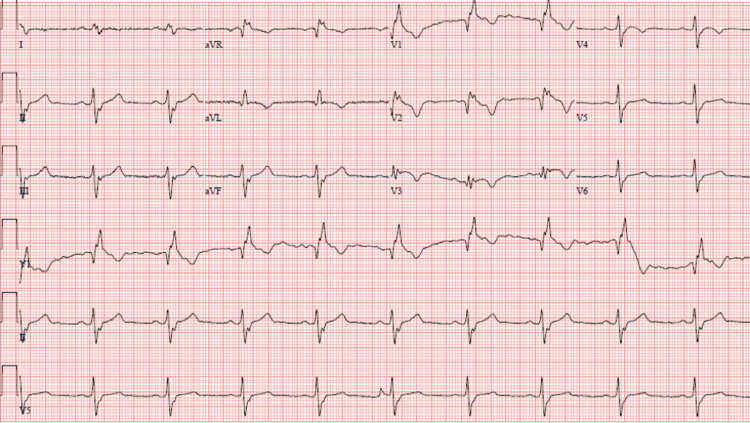
EKG revealing the patient’s baseline bifascicular block. EKG: electrocardiogram

A 51-year-old Caucasian male with no significant pulmonary or cardiac past medical history presented to the hospital complaining of sudden-onset dizziness, shortness of breath, and diaphoresis while walking up a flight of stairs. He denied chest pain and palpitations. Although he did not lose consciousness, the patient reported that he felt like he was about to. The patient denied having experienced similar symptoms in the past. Upon arrival to the emergency room, his vital signs were noted to be significant for only bradycardia, with a heart rate in the 50s. A 12-lead EKG revealed sinus bradycardia with a heart rate of 59 beats per minute and a baseline right bundle branch block and left anterior fascicular block (Figure 1).

**Figure 2:**
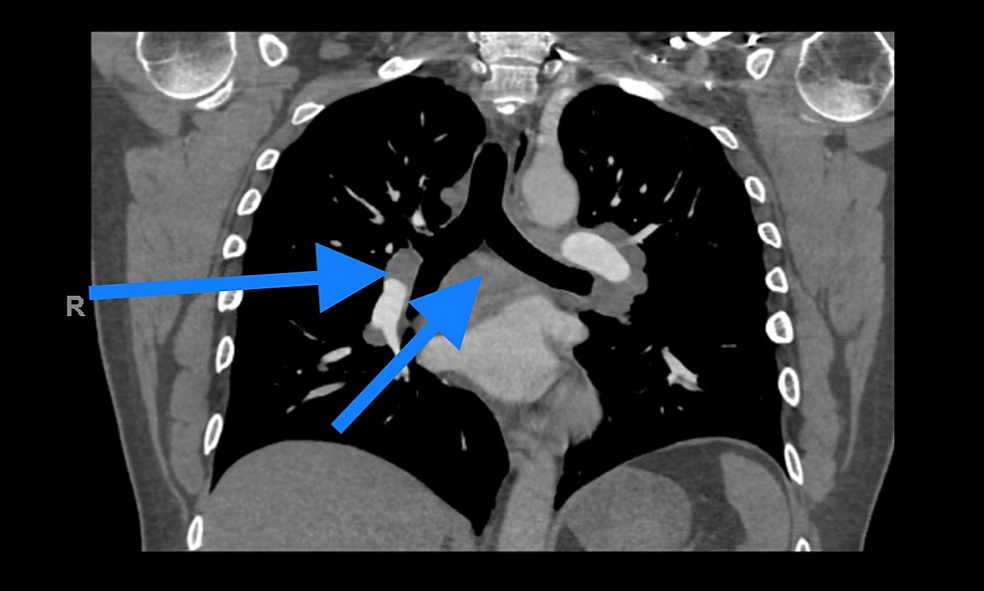
CT with IV contrast showing bilateral mediastinal and hilar lymphadenopathy. CT: computed tomography; IV: intravenous

Cardiac enzymes and thyroid function tests were normal. A chest X-ray did not show any acute processes. A subsequent computerized tomography (CT) with intravenous (IV) contrast of the chest, although unremarkable for pulmonary emboli, showed significant bilateral mediastinal and hilar lymphadenopathy (Figure 2).

Additionally, a transthoracic echocardiogram showed normal left ventricular function with an ejection fraction of 50-55% and no regional wall motion abnormalities. Due to the patient’s baseline bifascicular block and presyncopal symptoms, an electrophysiology study was completed which revealed normal sinoatrial and atrioventricular node function with inducible sustained ventricular tachycardia at a rate of 250 beats per minute. The patient continued to complain of intermittent light-headedness and dizziness at rest and with exertion; telemetry continued to capture sinus bradycardia with a heart rate in the 50s. Given the patient’s symptoms, the EKG and CT findings, and electrophysiology study results, suspicion for cardiac sarcoidosis was high at this point. Therefore, the patient underwent an endobronchial ultrasound-guided transbronchial needle aspiration of the mediastinal lymph nodes. A cardiac catheterization on hospital day five did not show significant atherosclerotic disease. Thereafter, a dual-chamber Boston Scientific defibrillator was placed for secondary prevention and the patient was discharged on metoprolol succinate 25 mg daily.

**Figure 3:**
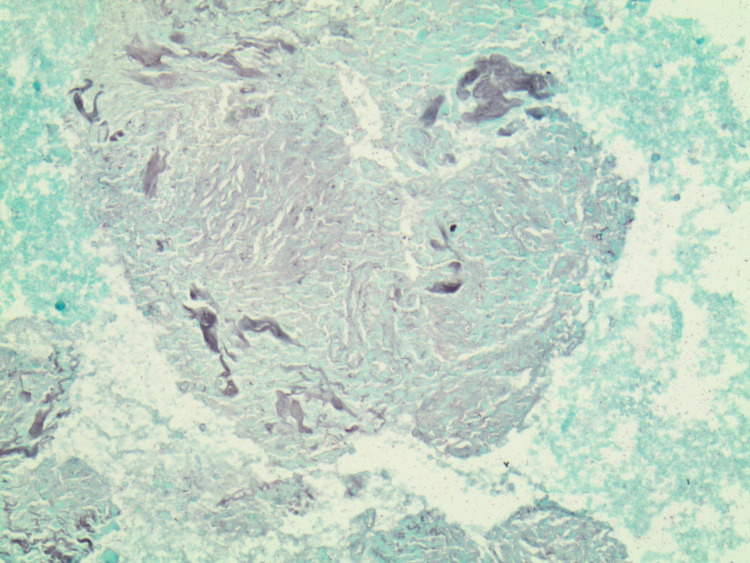
Histological slide from the EBUS-TBNA showing a noncaseating granuloma, which is a characteristic finding of sarcoidosis. EBUS-TBNA: endobronchial ultrasound-guided transbronchial needle aspiration

The pathology report of the mediastinal lymph nodes later confirmed the presence of noncaseating granulomas as seen in sarcoidosis (Figure 3) and the patient underwent an 18-fluorodeoxyglucose positron emission tomography (FDG-PET) scan to assess for myocardial involvement of the disease. The FDG-PET scan revealed findings suspicious for active sarcoidosis in the basal to the mid anterior and anterolateral wall, affecting approximately 24% of the left ventricular myocardium. Calcium, alkaline phosphatase, and 25-hydroxyvitamin D were found to be within a normal range.

The patient was started on 30 mg of prednisone daily along with 5 mg of methotrexate weekly in conjunction with daily folic acid supplements. The patient returned for a follow-up visit approximately three months after therapy was initiated and reported feeling asymptomatic. Subsequently, a decision was then made to taper prednisone to 10 mg every two weeks and to gradually increase methotrexate to 15 mg by 5 mg every week. A repeat FDG-PET scan done approximately six months after immunosuppression therapy was initiated showed improvement in disease activity, affecting only 6% compared to 24% of the left ventricular myocardium, and the patient remained asymptomatic.

## Discussion

Sarcoidosis, a multisystem granulomatous disease, most commonly involves the lungs [1]. In addition to being more prevalent in young and middle-aged adults, in the United States, African Americans have a three to four-fold greater risk for disease compared to Caucasians [3,7]. According to autopsy studies, at least 25% of patients with sarcoidosis are known to have cardiac involvement, yet only 5% of these patients exhibit clinically overt symptoms [1,4,5].

Diagnosis of cardiac sarcoidosis can be very challenging, largely due to nonspecific presenting symptoms [1-5,8,9]. The clinical presentation of the disease depends on the location and extent of granulomatous involvement and can vary from conduction abnormalities to ventricular arrhythmias and congestive heart failure [3]. Consequently, patient symptoms, or lack thereof, may also range tremendously. Our patient had near-syncopal symptoms, initially with exertion and later even while at rest, from active basal septum disease causing a bifascicular block.

A recent expert consensus statement on the diagnosis and management of cardiac sarcoidosis published by the Heart Rhythm Society stated that it is important to consider an electrophysiology study in patients with first-degree atrioventricular block or fascicular block to define the levels of conduction system disease, as was done for our patient [1]. An unexplained second-degree Mobitz type II or complete heart block and/or sustained monomorphic ventricular tachycardia of unknown etiology on electrophysiology studies should call for further investigation into the potential of cardiac sarcoidosis with a CT of the chest, two-dimensional echocardiography, contrast-enhanced magnetic resonance imaging (CMR), or FDG-PET [1]. Of these modalities, CMR and FDG-PET are the most sensitive, and findings seem to correlate with disease activity [3]. However, image interpretation can be challenging with both CMR and FDG-PET and must be made in the appropriate clinical context by an expert specialist [1]. The presence of noncaseating granulomas in tissue analysis, and the exclusion of other causes of granuloma formation, is the definitive test for organ involvement in sarcoidosis [1]. Lymph nodes or the lungs, due to a higher diagnostic yield and lower procedural risk, are typically targeted first to confirm the presence of noncaseating granulomas on pathology [1,4]. An endomyocardial biopsy (EMB) may be required to confirm the diagnosis if an extracardiac biopsy is negative or if isolated cardiac sarcoidosis is suspected [1,4]. However, according to consensus guidelines, due to the focal nature of the disease, EMB should be performed with imaging guidance or an electroanatomical map to increase sensitivity [1,4].

Although guidelines validated by prospective trials have not yet been established, the Heart Rhythm Society, the Japanese Ministry of Health and Welfare, and the National Institutes of Health have set forth criteria to aid in the diagnosis of cardiac sarcoidosis [8]. Assuming all other causes for the cardiac manifestations seen in a patient have been reasonably excluded, two pathways exist for diagnosing cardiac sarcoidosis. The first pathway involves obtaining a histological diagnosis from myocardial tissue, and the second pathway involves diagnosing “probable” cardiac sarcoidosis based on invasive and noninvasive studies [5]. The aforementioned second pathway was used for our patient; otherwise, unexplained induced ventricular tachycardia was evident on electrophysiology study, FDG-PET revealed a disease pattern consistent with cardiac sarcoidosis, a histologic diagnosis of extracardiac sarcoidosis was made, other reasonable causes of the patient’s presentation were excluded, and lastly, the patient exhibited clinical and radiographical improvement in disease activity with immunosuppressive therapy.

The clinical efficacy and optimal initial dose and duration of corticosteroid treatment of cardiac sarcoidosis remain controversial. Although the exact mechanism of action of steroids in treating cardiac sarcoidosis is unknown, steroids are believed to slow the progression of inflammation and fibrosis [3]. A recent study indicated that the effects of an initial high dose (>40 mg daily) did not differ from those of a low dose (30 mg or less daily) [10]. Therefore, initiating therapy with 30 to 40 mg of prednisone with re-evaluation after one to three months is suggested [4,5,8]. If a patient is responding clinically and demonstrating improvement on cardiac imaging results, a subsequent dose adjustment and/or tapering by 5 to 10 mg per day with treatment for an additional nine to twelve months is recommended [4,5,8,11]. Alternative agents such as methotrexate have also been reported to be effective in treating cardiac sarcoidosis, and are recommended to be started early in the treatment course along with steroids or in situations where the disease is refractory to steroid treatment or if the patient is unable to tolerate chronic steroid use [9,12]. Due to the risk of relapse, it is recommended that patients continue to be monitored for disease recurrence for at least three years [11]. Our patient responded well to a starting dose of prednisone 30 mg daily and methotrexate 5 mg weekly. Given that the patient was asymptomatic three months after the therapy was initiated, a decision was made to taper prednisone by 10 mg every two weeks and to increase methotrexate to 15 mg incrementally. The FDG-PET obtained approximately six months after therapy initiation validated the patient’s clinical improvement.

Additionally, although steroids may improve survival in a patient diagnosed with cardiac sarcoidosis, they do not reduce the incidence of ventricular arrhythmias or sudden cardiac death. Thus, it is important to consider internal cardioverter-defibrillator (ICD) placement in patients with sarcoidosis and a history of inducible sustained ventricular tachycardia [1]. To date, no study has evaluated the use of antiarrhythmic agents in cardiac sarcoidosis [1]. Our patient had an ICD implanted as secondary prevention and was placed on metoprolol succinate 25 mg daily. Device interrogation done in the immediate follow-up period after placement did not reveal the presence of any episodes of ventricular tachycardia.

Patients with cardiac sarcoidosis tend to have a more guarded prognosis compared to those without cardiac involvement [8]. Additionally, in patients with symptomatic cardiac sarcoidosis, the extent of left ventricular dysfunction is an important indicator of survival [8,13]. Due to the advanced pharmacological and device therapy available to treat heart failure, the major cause of death in patients with cardiac sarcoidosis can be attributed to sudden cardiac death from ventricular arrhythmias [8]. The prognosis of clinically silent cardiac sarcoidosis remains controversial [1,4,5,8].

## Conclusions

To limit morbidity and mortality, as evidenced in our case, it is important for clinicians to quickly differentiate between benign and potentially life-threatening causes of syncope. Although rare, presyncope or syncope may be the initial clinical manifestation of underlying cardiac sarcoidosis. Suspicion for the disease should remain high when encountering such patients who also have unexplained baseline EKG abnormalities.
